# Integrating Survey and Molecular Approaches to Better Understand Wildlife Disease Ecology

**DOI:** 10.1371/journal.pone.0046310

**Published:** 2012-10-05

**Authors:** Brendan D. Cowled, Michael P. Ward, Shawn W. Laffan, Francesca Galea, M. Graeme Garner, Anna J. MacDonald, Ian Marsh, Petra Muellner, Katherine Negus, Sumaiya Quasim, Andrew P. Woolnough, Stephen D. Sarre

**Affiliations:** 1 Faculty of Veterinary Science, The University of Sydney, Camden, Australia; 2 The School of Biological, Earth and Environmental Sciences, The University of New South Wales, Sydney, Australia; 3 Department of Primary Industries NSW, Elizabeth Macarthur Agricultural Institute, Camden, Australia; 4 Office of the Chief Veterinary Officer, Department of Agriculture, Fisheries and Forestry, Canberra, Australia; 5 Institute for Applied Ecology, University of Canberra, Canberra, Australia; 6 Epi-Interactive, Wellington, New Zealand; 7 Victorian Department of Primary Industries, Melbourne, Victoria, Australia; University College Dublin, Ireland

## Abstract

Infectious wildlife diseases have enormous global impacts, leading to human pandemics, global biodiversity declines and socio-economic hardship. Understanding how infection persists and is transmitted in wildlife is critical for managing diseases, but our understanding is limited. Our study aim was to better understand how infectious disease persists in wildlife populations by integrating genetics, ecology and epidemiology approaches. Specifically, we aimed to determine whether environmental or host factors were stronger drivers of *Salmonella* persistence or transmission within a remote and isolated wild pig (*Sus scrofa*) population. We determined the *Salmonella* infection status of wild pigs. *Salmonella* isolates were genotyped and a range of data was collected on putative risk factors for *Salmonella* transmission. We *a priori* identified several plausible biological hypotheses for *Salmonella* prevalence (cross sectional study design) versus transmission (molecular case series study design) and fit the data to these models. There were 543 wild pig *Salmonella* observations, sampled at 93 unique locations. *Salmonella* prevalence was 41% (95% confidence interval [CI]: 37–45%). The median *Salmonella* DICE coefficient (or *Salmonella* genetic similarity) was 52% (interquartile range [IQR]: 42–62%). Using the traditional cross sectional prevalence study design, the only supported model was based on the hypothesis that abundance of available ecological resources determines *Salmonella* prevalence in wild pigs. In the molecular study design, spatial proximity and herd membership as well as some individual risk factors (sex, condition score and relative density) determined transmission between pigs. Traditional cross sectional surveys and molecular epidemiological approaches are complementary and together can enhance understanding of disease ecology: abundance of ecological resources critical for wildlife influences *Salmonella* prevalence, whereas *Salmonella* transmission is driven by local spatial, social, density and individual factors, rather than resources. This enhanced understanding has implications for the control of diseases in wildlife populations. Attempts to manage wildlife disease using simplistic density approaches do not acknowledge the complexity of disease ecology.

## Introduction

Infectious diseases of wildlife have caused important global pandemics in people [1], have influenced human welfare through reduced agricultural production [2], [3] and are reducing global biodiversity [4]–[6]. Management of wildlife infectious disease requires that key ecological processes and mechanisms that drive infection transmission and persistence in wildlife populations be identified, characterised and quantified [7], [8]. Despite this, little is known about disease transmission in wildlife [9]. Improved knowledge could assist management of wildlife disease thereby reducing disease emergence that threatens human and animal health and welfare, agricultural production and species conservation. In this respect, Daszak et al. [10] have proposed that emerging infectious diseases of wildlife can be classified into three major groups based on key epidemiological criteria – spillover from domestic animals to wildlife populations living in proximity; those related directly to human intervention via host or parasite translocations; and those with no overt human or domestic animal involvement.

Cross sectional surveys – where a representative sample of a population is taken, prevalence of disease measured and a contrast made between those with and without the infection to infer risk factors – have frequently been a mainstay of wildlife epidemiological study [11]. However, in recent years it has been recognised that the interface of genetics, ecology and epidemiology is poised to advance our understanding of disease ecology, such as infection transmission, in new and novel ways [12], [13].

Here, we use both the traditional and widely accepted cross sectional survey design to investigate risk factors for prevalence or persistence of *Salmonella* and a contemporary molecular epidemiological study design to assess risk factors for transmission of *Salmonella* in wild pigs. We use wild pigs (*Sus scrofa*) as our model as they are a species of global importance, being found on every continent except Antarctica [14]. They are a damaging invasive species or valued endemic animal depending on their location, and have frequently played an important role in the spread of infectious diseases [15]–[19], including *Salmonella* [20]. There are two *Salmonella* species, six subspecies and more than 2500 serovars [21]. In this paper *Salmonella* refers to serovars within *Salmonella enterica* subspecies *enterica*.

## Materials and Methods

We sampled [22] and determined the *Salmonella* infection status of 543 wild pigs (*Sus scrofa*) by culturing mesenteric lymph nodes and faeces [23]. *Salmonella* isolates were genotyped using Pulsed field gel electrophoresis (PFGE) [24]. A range of data were collected on putative risk factors for *Salmonella* transmission including: pig genotype (using microsatellites) [25], spatial and remote sensing data [26] and pig demographic and morphological data [25], [27]. We took two approaches to analyse the data.

### 1. Cross sectional prevalence study design

A traditional cross sectional prevalence based logistic regression analysis which modelled associations between *Salmonella* infection and risk factors.

### 2. Molecular case series approach

A pair-wise molecular analysis of all *Salmonella* isolates which modelled *Salmonella* genetic relatedness for each pair of infected pigs against risk factors using linear regression. It was assumed that increasing similarity between *Salmonella* isolates was correlated with transmission.

We *a priori* identified several plausible biological hypotheses for both *Salmonella* prevalence in pigs (cross sectional prevalence study design) and *Salmonella* transmission between pigs (molecular case series study design). Two models (paired) were implemented for each hypothesis, one for the cross sectional prevalence study design and one for the molecular case series study design. We used information theoretic approaches to select the most supported models within each study design. We compared the two study design for utility for wildlife disease investigation and inferred mechanisms for *Salmonella* persistence and transmission between wild pigs.

**Figure 1:**
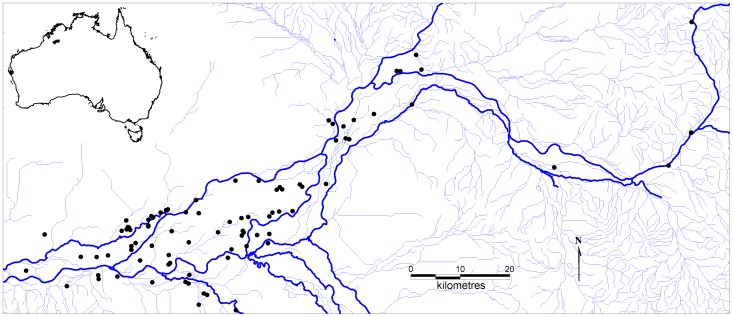
The study area. The inset shows the study area in the context of the Australian continent. The solid and dashed lines represent major and minor drainage lines respectively. The dots represent sampling locations.

The study was undertaken on a 200 km length of the Fitzroy River floodplain in the west Kimberley region of north-western Australia (see Figure 1) in October 2010. The study area is approximately 4000 km^2^, lying between 18.612 to 18.037°S and 124.922 to 126.270°E. The area has a tropical monsoonal climate with mean rainfall over the last century of 484 mm per year (range 163–907) and hot temperatures (mean daily maximum temperature 30–39°C [range 18–46°C]).The sampling strategy was to systematically search water features by helicopter. All pigs observed were targeted for humane destruction according to Australian standard operating procedures [22]. Dead pigs were sampled within one hour by veterinarians. Faeces and mesenteric lymph nodes (MLN) were collected, immediately refrigerated and delivered to the laboratory chilled for culture. A sample of ear tissue was also collected from each pig and stored in salt-saturated DMSO buffer for genetic analysis.

Faeces and MLN were cultured according to the Australian standard for microbiology of food and animal feeding stuffs for the detection of Salmonella spp. [23] which is a national modification of the international standard (ISO 6579:2002). All *Salmonella* isolates confirmed by serotyping were genotyped by PFGE [24]. Gel images were imported into BioNumerics (version 6.6) for data analysis [28]. All gels were normalised using the *S. Braenderup* reference strain. Bands were detected automatically and verified manually. *Salmonella* PFGE DICE similarity coefficient was extracted for each pair-wise comparison of isolates using optimised parameters (optimisation (0.2%), tolerance (1.375%), tolerance change (0%), minimum height (9%), minimum surface (2%), uncertain bands (ignore), relaxed doublet matching (no), fuzzy logic (no), area sensitive (no) and active zones (4.3–88.6%)). DNA was extracted from pig ear tissues using the Machery Nagel NucleoSpin Tissue kit. Fourteen pig microsatellite markers [25] were amplified from DNA samples in three multiplex PCRs using the Qiagen Multiplex PCR kit. Microsatellites were genotyped on a Beckman CEQ8000 and alleles were scored using the CEQ 8000 Genetic Analysis System software Version 8.0.

Two data sets were assembled. The first data set (for the cross sectional prevalence study design) consisted of a single observation for each pig that was sampled. Presence or absence of infection was determined by culture for each pig in this dataset. Covariate data (as described in Table 1) were collected for assessment as risk factors or extraneous variables. The second data set (molecular case series study design) was a subset of the first and consisted of pair-wise comparisons between *Salmonella* isolates (case series with no counterfactual group). The outcome variable was the pair-wise genetic similarity (DICE) for each pair of *Salmonella* isolates. Covariates were the same as for the first data set except that they were the absolute difference between pair-wise covariates values, pair-wise contrasts, for example Euclidean distance between pigs, or pair-wise indicator variables (for example to indicate the same herd membership).

**Table 1:** Data description and hypotheses for *Salmonella* persistence and transmission in wild pigs (*Sus scrofa*) modelled using both the cross sectional prevalence study design and molecular case series study design.

Hypothesis	Rationale
Density of hosts	Numerous authors have presented transmission models [40], [47]. Increasingly, there is evidence that systems are more complex and that density-dependence does not hold [40], [48]. Other research has indicated that endemic marsupials near the study area have a high prevalence and diversity of *Salmonella* [49]. Here we hypothesised that increasing local or larger scale densities of wild pigs and native species (*Macropus agilis*) would increase prevalence and transmission of *Salmonella*. We therefore modelled *Salmonella* infection or *Salmonella* genetic similarity (DICE) against group size and density of groups of wild pigs and density of groups of agile wallabies (groups km^−2^) as measured during an aerial survey of pigs and agile wallabies in the study area using published aerial survey methodology [50].
Environmental contamination	*Salmonella* survives in the environment after defecation by pigs for considerable periods of time especially in cooler conditions away from light [51]–[53]. Our environmental persistence hypothesis assumed that environmental contamination from pig defecation was responsible for *Salmonella* prevalence or transmission and that areas of shade such as trees would increase *Salmonella* prevalence or transmission. We modelled *Salmonella* infection or *Salmonella* genetic similarity (DICE) against the mean EVI [26] for the 6 months prior to sampling in each pigs home range (radius of approximately 2.5 km around each sampled pig). The distance to water resources, number of water bodies within the home range, density of pig herds, pig herd size and densities of the agile wallaby (*Macropus agilis*) were included as covariates to allow conditioning on these covariates for control of confounding.
Host immunity	There are many reasons to assume that individual animal factors such as sex, age and dominance can affect prevalence of infection [11]. We hypothesised these factors affected *Salmonella* prevalence or transmission and modelled age [38], condition score [36] and gender against *Salmonella* infection and *Salmonella* genetic similarity (DICE).
Resources	Under the hot semi arid conditions of the study area, critical resources for wild pigs are water [54], riverine habitat for thermal protection [55] and plant food resources [54], [56]. Pigs are highly social animals [23] that do not establish and defend territories [54], and as such have overlapping home ranges [22]. Distribution across a landscape therefore largely depends on resource availability. Thus, we modelled *Salmonella* infection or *Salmonella* genetic similarity (DICE) against features likely to cause aggregation of pigs (Euclidean distance to major water courses (m), the number of natural water-bodies in a pig home range and pasture availability (change in EVI over 6 months)) but included density, size of social group and condition score to control confounding.
Social interaction	We assumed that pigs that were more related would have greater physical contact and hence transmit *Salmonella* to one another more readily. We regressed pair-wise *Salmonella* genetic similarity [38] against pair-wise genetic distance of pigs [41] (derived from microsatellites [36]), controlling for common herd occupancy and Euclidean distance to prevent confounding. We could not assess this in the prevalence approach as little genetic structuring was detected in pigs across the study area (data not shown) and pairwise comparisons were not possible.

Five biologically plausible hypotheses were developed *a priori* that sought to explain the prevalence or transmission of *Salmonella* between pigs within the study area (see Table 1). Two separate models were then developed for each hypothesis, one model for the cross sectional prevalence study design and one for the molecular case series study design. For the simple presence and absence data set, multivariable logistic regression models were developed, in which the covariates were related to the log odds of infection with *Salmonella* (i.e. generalised linear models with a logit link). For the pair-wise data set, multivariable linear models were developed that related *Salmonella* DICE linearly to pair-wise covariates. Permutation based methods (999 permutations [29]) were used to assess the significance of coefficients in the linear models, with coefficients ranked amongst the highest 2.5% or lowest 2.5% of permutation coefficients being considered significant at α = 0.05. Examination of the relative strength of evidence for each hypothesis within either the prevalence or molecular approach was undertaken using the approach of Burnham and Anderson [30]. The cross sectional prevalence and molecular case series study designs were then compared for consistency of outcome and relative utility. Inferences for risk factors for *Salmonella* prevalence (persistence) or transmission were made.

This study was approved by the University of Sydney Animal Ethics Committee (N00/6-2010/1/5319).

## Results

We made 543 wild pig *Salmonella* observations, sampled at 93 unique locations across the study area. The mean weight of pigs was 51 kg (95% CI: 47–54) with a range of 2–150 kg. There were 264 males (49%) and 279 females (51%). Mean male weight was slightly greater than female weight 54 (95% CI: 50–58) versus 49 (95% CI: 45–52) kg, respectively. Assuming an adult wild pig is ≥30 kg [21], there were 361 adults (66%) and 182 sub adults (34%). Of the adult females, 58 were pregnant and 76 were lactating. The prevalence of *Salmonella* infection was 41% (95% CI: 37–45%). The median *Salmonella* DICE coefficient [28] (or *Salmonella* genetic similarity) was 52% (IQR: 42–62, range: 10.0–100). The median pig genetic dissimilarity [31] was 39% (IQR: 35–46, range: 7–71).

The only model supported using the traditional cross sectional prevalence study design, represented the hypothesis that the abundance of available ecological resources is associated with *Salmonella* infection in wild pigs (Table 2). All other hypotheses were highly unlikely. In the contemporary molecular study design assessing risk factors for transmission, the resource driven contact and host immunity models were equally supported models, with other models/hypotheses being unsupported by the data (Table 3).

**Table 2:** Akaike information criterion (AIC) values and other model selection metrics for cross sectional prevalence logistic regression models using information theoretic approaches [29].

Model	Parameters (K)	Bias corrected AIC (AICc)	AICc differences (Δ)	Relative likelihood (evidence ratio)	Probability (Akaike weight)
Resource driven contact	10	699.8	0.0	1.0	0.994
Environmental contamination	8	710.9	11.1	251.7	0.004
Density dependant	6	712.1	12.2	455.6	0.002
Host immunity	6	713.6	13.7	964.5	0.001

The probability of the resource transmission model is very high (>0.99) and clearly the data support this model. Models are listed in AIC ranked order for each study design.

**Table 3:** Akaike information criterion (AIC) values and other model selection metrics for molecular case series linear models using information theoretic approaches [29].

Model	Parameters (K)	Bias corrected AIC (AICc)	AICc differences (Δ)	Relative likelihood (evidence ratio)	Probability (Akaike weight)
Host immunity	6	339132.1	0.0	0.98	0.580
Resource driven contact	11	339132.7	0.6	1.0	0.420
Environmental contamination	8	339218.0	85.9	4.4×10^18^	0.000
Genetic relatedness model	5	339284.7	152.6	1.4×10^33^	0.000
Density dependant	7	339735.4	603.3	1.0×10^131^	0.000

The probability of both the resources and host immunity models is high rather than the other hypothesised mechanisms of transmission. Models are listed in AIC ranked order for each study design.

Significant coefficients in the cross sectional prevalence data resource model (Table 4) indicated that resources such as the change in enhanced vegetation index (EVI) over the monsoon season (representing areas where pasture growth is marked during the wet season) and proximity to waterways (representing water to drink and riparian zone shelter) were critical features that were associated with increasing prevalence of infection. Additionally, better conditioned (fatter) pigs and increasing wild pig density (weakly: P = 0.04) were associated with increasing probability of infection (Table 4). In the contemporary molecular study design assessing risk factors for transmission, the resource driven contact (Table 5) and host immunity (Table 6) models were equally supported models, with other models/hypotheses being unsupported by the data. However, the only significant coefficients from the resources model were the extraneous variables included to control confounding of the association between *Salmonella* genetic similarity and the resource covariates. Thus it was apparent that resource availability had little role in transmission. Interpretation of significant coefficients for the indicator variables from the two models demonstrated that transmission of *Salmonella* was increased between members of the same herd and between males relative to other sexes, whilst controlling for isolates that came from the same individual (isolates from the same individual were more similar as expected). Transmission was more likely between pigs that were geographically closer with the similarity of *Salmonella* declining as the Euclidean distance between pigs increased. Interpretation of the remaining significant covariates from the resources and host immunity models was more complex as these covariates were the absolute differences between the value of the covariate for each pig being compared, and are thus undirected associations. There was an association between *Salmonella* similarity and divergent ages and densities of source population of the pigs being compared. The coefficient for condition score indicated that transmission was less likely between pigs of differing condition score.

**Table 4:** Resource hypothesis model formulation and coefficient estimates for cross sectional prevalence study design.

Model	Parameter	Coefficient estimate	Standard error	Z value	P value	Odds ratio
log [π÷(1−π)] = β_1_+β_2_CS+β_3_DS+β_4_ |ΔEVI|+β_5_HS+β_6_NWB+β_7_DW+β_8_DP+β_9_X+β_10_Y+r.eff.(location) Calibration: le Cessie-van Houwelingen goodness of fit test (Z = 0.2, P = 0.8) Validation: AUC 0.7 Pseudo r^2^ = 14%	(Intercept)	12.04	143.76	0.08	0.93	…
	**Condition score (CS)**	**0.76**	**0.39**	**1.93**	**0.05**	**2.13**
	**Dist. to streams (DS)**	**−0.57**	**0.21**	**−2.73**	**0.01**	**0.57**
	**EVI decline (ΔEVI)**	**−0.38**	**0.15**	**−2.59**	**0.01**	**0.68** *
	Herd size (HS)	0.01	0.02	0.61	0.54	1.01
	No. water bodies (NWB)	0.06	0.05	1.26	0.21	1.06
	Wallaby herd density (DW)	0.38	0.38	1.00	0.32	1.46*
	**Wild pig density (DP)**	**−0.84**	**0.40**	**−2.09**	**0.04**	**0.43** *
	X coordinate (X)	−0.34	0.90	−0.38	0.71	0.71
	Y coordinate (Y)	−1.60	2.29	−0.70	0.49	0.20

Random effects terms for herd, and fixed effect covariates for latitude and longitude were included to control clustering of data and spatial trends or autocorrelation.

* These covariates were transformed (normalised z = (x−μ)÷σ) to yield more interpretable odds ratios.

**Table 5:** Resource hypothesis model formulation and coefficient estimates for molecular case series study design.

Model	Parameter	Coefficient estimate	Standard error	Z value	P value
*Salmonella* DICE = β_1_+β_2_|CS|+β_3_|DS|+β_4_ ED+β_5_|ΔEVI|+β_6_|HS|+β_7_|DW|+β_8_|WB|+β_9_H+β_10_Pig+β_11_|DP| Adjusted r^2^ = 3%	**(Intercept)**	**55.55**	**0.25**	**224.19**	**0.001**
	**|Condition score| (|CS|)**	**−2.99**	**0.32**	**−9.48**	**<0.001**
	|Dist. to streams| (|DS|)	−0.22	0.16	−1.33	0.098
	**Euclidean distance (ED)**	**−0.02**	**0.00**	**−4.53**	**<0.001**
	|EVI decline| (|ΔEVI|)	0.00	0.00	−0.46	0.303
	|Herd Size| (|HS|)	−0.02	0.01	−1.31	0.107
	**|Wallaby density| (|DW|)**	**−3.20**	**0.40**	**−8.07**	**<0.001**
	|water bodies| (|WB|)	0.07	0.06	1.29	0.088
	**Same herd (Indicator: 0 = false) (H)**	**1.14**	**0.24**	**4.71**	**0.001**
	**Same pig (Indicator: 0 = False) (Pig)**	**44.45**	**2.00**	**22.27**	**<0.001**
	**|Wild pig density| (|DP|)**	**16.41**	**2.67**	**6.15**	**<0.001**

A fixed effect (indicator) covariate for herd and the distance between two pigs were included to control clustering and spatial autocorrelation.

**Table 6:** Host immunity hypothesis model formulation and coefficient estimates for molecular case series study designs.

Model	Parameter	Coefficient estimate	Standard error	Z value	P value
*Salmonella* DICE = β_1_+β_2_|Age|+β_3_|CS|+β_4_ED+β_5_H+β_6_Pig+β_7_Sex Adjusted r^2^ = 2%	**(Intercept)**	**56.81**	**0.26**	**216.33**	**<0.001**
	**|Age|**	**−0.58**	**0.00**	**−3.01**	**0.001**
	**|Condition score| (|CS|)**	**−3.01**	**0.31**	**−9.57**	**<0.001**
	**Euclidean distance (km) (ED)**	**−0.03**	**0.00**	**−11.39**	**<0.001**
	**Same herd (Indicator: 0 = false) (H)**	**0.71**	**0.23**	**3.04**	**0.001**
	**Same pig (Indicator: 0 = False) (Pig)**	**44.45**	**1.99**	**22.32**	**0.001**
	**Sex Indicator variable (reference Male: Male) (Sex)**				
	**Female/Female**	**−1.89**	**0.24**	**−7.88**	**<0.001**
	**Female/Male)**	**−0.92**	**0.22**	**−4.19**	**<0.001**

A fixed effect (indicator) covariate for herd and the distance between two pigs were included to control clustering and spatial autocorrelation.

## Discussion

It is interesting to posit the reasons that *Salmonella* prevalence was higher in well conditioned (fatter) pigs and in resource rich areas across the landscape. Prevalence of an infection in a population depends on several factors, especially transmission rates, but also disease induced mortality, duration of infection and the length of time an infection has been present in a population [11]. Given that resources were not observed to be important to transmission in the molecular approach, it is likely that wild pigs in resource rich areas may have had higher prevalence for reasons other than transmission. The last of these alternate explanations above, namely that prevalence is increased in populations where infection has been present in populations for longer is intriguing. It suggests that resource rich areas across the landscape may act as areas for persistence of *Salmonella* in pig populations. With regards to condition score as a risk factor, it is possible that those pigs that were fatter exhibited some specific behaviour that increased exposure to infection. Given their better body condition, these pigs may have been travelling further and foraging more effectively and widely for food. This may have exposed them to more infection, resulting in higher prevalence in these pigs through more effective contacts. This is consistent with prior research in human infections [32], and recently demonstrated in wildlife [33] that super-spreaders (or individuals responsible for the majority of transmission) are disproportionately important for disease transmission. However, reverse causality concepts associated with cross sectional surveys also suggests alternate explanations, such as that better conditioned pigs simply survived better with *Salmonella* infection.

The molecular study design demonstrated that transmission was more likely within social groups and to other pigs within close proximity. This was expected as wild pigs have been shown to be generally very sedentary [34], highly social [35] and with overlapping home ranges [36]. This suggests the mechanism for transmission is largely social and foraging behaviour between local pigs that increases effective contact. Transmission was also more common between males. Adult male pigs have larger home ranges than females [34] and are often found singly or associating in small male groups in the study area [37]. These characteristics may account for the observed greater male to male transmission. It also suggests that older males may be relatively more important in the transmission of *Salmonella* in our study area.

An important result from the molecular study design were the low R^2^ values associated with both the risk and resources model, indicating the majority of the variability in the *Salmonella* genetic relatedness was not due to direct transmission between wild pigs. Instead complex phylodynamic processes [38] or the presence of other species involved in *Salmonella* ecology likely introduced considerable *Salmonella* genetic diversity that we could not model under the assumption of transmission between pigs. Phylodynamic processes include mechanisms associated with the persistence of *Salmonella* infections within individual pigs, leading to increased selection pressure and interplay between host immune responses and mutations [38]. Were we to rely only on the traditional cross sectional study design and assume that infection status was correlated with infection transmission we would have overestimated the direct transmission between pigs that occurred in our study population, with the R^2^ value of the cross sectional resources model an order of magnitude greater than the resources or risk model in the molecular study. Additionally, an assumption that transmission was associated with resources would have been made, instead of the likely reason that resources affected prevalence through other reasons such as persistence of *Salmonella* in pig populations in resource rich areas.

Our empirical findings, that *Salmonella* persistence in pig populations is associated with resource abundance, and conversely that density has little role in persistence, have implications for control of infection in pigs in Northern Australia and in wildlife more generally. It indicates that control of wildlife infections may not always be achieved through simplistic application of threshold density concepts, as indicated by prior theoretical models [39], [40]. Based on this principal, many authors have proposed that simply reducing abundance or the susceptible proportion of wildlife (e.g. by culling or vaccination) will lead to disease fadeout because transmission cannot be maintained [41]. Other authors argue that empirical evidence of such an effect is lacking [42], [43], or that percolation thresholds better explain empirical data of threshold densities [43]. Our results do not disprove a threshold effect but do indicate that targeting areas for control simplistically based on density may not be a useful strategy. Instead careful consideration of resource distribution across the landscape and spatial targeting of control to those areas of greatest risk would be more efficient at reducing prevalence than control targeted at wildlife density alone. Additionally, the local transmission observed in our study suggests that in the event of a spreading epidemic in a naive population where vaccination or culling zones are implemented [44], these zones can be structured on the probable movements of local pigs (especially males). Transmission was also more likely between pigs in areas where the density of their local population differed markedly. The role of density in control may thus be to allow targeting of areas where transmission may occur from population to population (i.e. at areas of divergent density).

*Salmonella* is an important human pathogen. It is also important in livestock production, both due to its effect on health and productivity and due to its role as a foodborne zoonosis. *Salmonella* has been isolated from the carcasses of wild pigs harvested for human consumption in Australia [45], [46]. *Salmonella* infection of wild pig populations might represent a reservoir of infection for grazing livestock (sheep and cattle) or pose a direct (wild pigs are hunted as a recreational pursuit) and a foodborne (wild pigs are commercially harvested) zoonotic hazard [35]. The role that wild pig populations might play as reservoirs of *Salmonella* for domestic livestock apparently has not been investigated. Whether this is an ecosystem with no overt domestic animal (or human) involvement i.e. wild pigs as a reservoir, or spillover from domestic animals to wildlife populations living in proximity [10], is an open question. In the current study evidence was found to support ecological resources as a driver of Salmonella transmission; in addition, spatial proximity and other host factors influenced transmission between pigs. These results suggest that a Salmonella pig-pig ecosystem exists but does not answer the open question posed above. However, we have commenced research to determine whether the wild pig population described in this study is a reservoir of *Salmonella* for co-grazing cattle by using molecular and spatial epidemiological methods.

We conclude that molecular epidemiological approaches and traditional cross sectional surveys are complementary and can enhance the understanding that can be achieved using either approach alone. Even in a complex hyper-endemic *Salmonella* ecological system, strong signals were evident and greater inferences were possible than using either approach alone. Our analyses indicated that the abundance of ecological resources critical for wildlife influences *Salmonella* prevalence, likely through greater persistence of *Salmonella* in wild pig populations. Importantly the use of a molecular approach allowed differentiation between persistence and transmission of *Salmonella*, revealing that transmission is influenced by local spatial, social and individual factors, rather than just resources. Additionally, reliance on only cross sectional data for evaluating *Salmonella* transmission would have overestimated the proportion of variability of *Salmonella* data that could be explained, with R^2^ values orders of magnitude greater than with the molecular approaches. The integration of molecular and cross sectional approaches also allows nuanced inferences for control. Implementation of control zones for wildlife disease management should be structured on complex spatial, social, density and resource distribution principals that aim to reduce prevalence as well as transmission, rather than on simple host density principals outlined in previous theoretical models.
